# Donor-conceived adolescents’ interest in obtaining information about their identity-release oocyte or sperm donor—results from the Swedish Study on Gamete Donation

**DOI:** 10.1093/humrep/deag088

**Published:** 2026-05-27

**Authors:** Henrik Groundstroem, Johan Paulin, Emilia Thorup, Gunilla Sydsjö, Claudia Lampic

**Affiliations:** Department of Psychology, Umeå University, Umeå, Sweden; Department of Psychology, Umeå University, Umeå, Sweden; Department of Occupational Health, Psychology and Sports Science, University of Gävle, Gävle, Sweden; Department of Psychology, Lund University, Lund, Sweden; Division of Obstetrics and Gynaecology, Department of Clinical and Experimental Medicine, Faculty of Health Sciences, Linköping University, Linköping, Sweden; Department of Psychology, Umeå University, Umeå, Sweden

**Keywords:** donor conception, oocyte donation, sperm donation, identity-release, adolescent

## Abstract

**STUDY QUESTION:**

Do donor-conceived adolescents in lesbian- and heterosexual-couple families intend to request information about their identity-release oocyte or sperm donor?

**SUMMARY ANSWER:**

While more than half of adolescents intended to request donor information, being uncertain about making such requests was quite common, particularly among adolescents reporting better family functioning and those conceived with donor sperm in heterosexual-couple families.

**WHAT IS KNOWN ALREADY:**

Increasing numbers of children conceived with identity-release gamete donation are approaching the age when they can obtain donor information, but there is very little knowledge about their perspectives regarding this possibility, particularly among those conceived with donor oocytes.

**STUDY DESIGN, SIZE, DURATION:**

The present cross-sectional survey study was conducted 2022-2023 as part of a longitudinal follow-up of lesbian- and heterosexual-couple families following identity-release sperm donation (SD-L, SD-H) or oocyte donation (OD-H). We approached parents in a total of 216 families with a survey and also requested their consent to contact their donor-conceived child, provided he/she was aware of the donor conception. Parents in 165 families participated in this follow-up and, among these, parents in 123 families (75%) also provided contact information to their child(ren). Out of 128 approached adolescents, 100 completed the survey (78% response rate). Following exclusion of four adolescents with known/directed donors, the study sample consisted of 96 donor-conceived children aged 13–16 years (47 SD-L, 27 SD-H, and 22 OD-H), most of whom had been aware of their donor conception from an early age.

**PARTICIPANTS/MATERIALS, SETTING, METHOD:**

The present study was based on the fifth wave of data collection of the Swedish Study on Gamete Donation, which used consecutive recruitment of recipient couples starting donation treatment 2005-2008. In Sweden, recipients of donor gametes receive no information about the donor. Only the donor-conceived person has the legal right, when sufficiently mature, to request information about the donor, including his/her identity. Study-specific items were used to assess adolescents’ experiences and perceptions of disclosure issues, interest in requesting any donor information (identifying and/or non-identifying) as well as interest in contact with the donor and same-donor peers. Family relationships were assessed with the FAD GF6+ and study-specific items measuring perceived parent-child closeness and resemblance. Comparisons between the three donation groups (SD-L, SD-H, OD-H) were performed with χ^2^ test and Fisher exact test. Multinomial logistic regression analysis was performed in two separate models to investigate if the intention to request donor information was associated with family type (lesbian/heterosexual-couple) and with donation type in heterosexual-couple families (SD-H/OD-H).

**MAIN RESULTS AND THE ROLE OF CHANCE:**

More than half (58%) of adolescents intended to request information about the donor (as soon as possible or sometime in the future), while 1 in 10 had no such intention, and a third of adolescents were uncertain. Regression analysis showed no significant effect of family form (lesbian/heterosexual-couple) on the intention to request donor information, but better family functioning (FAD GF6+) was associated with being uncertain about making such requests (*P *= 0.036) (OR 0.19, 95% CI 0.04–0.90). The second regression analysis, restricted to adolescents from heterosexual-couple families, showed that those from SD-H families were more likely than OD-H adolescents to be uncertain about requesting donor information (*P *= 0.028) (OR 0.17, 95% CI 0.04–0.82), when controlling for gender, family functioning, and closeness with the non-genetic parent. The most common motivations for intending to request donor information were to look for resemblance to the donor and to learn about one’s heritage, and one in five was motivated by a desire to contact the donor. The adolescents wanted to receive information about the donor’s background (78%), identity (61%), and openness to being contacted (52%), with no significant differences between donation groups. In addition, a third of the adolescents were interested in contact with same-donor peers, irrespective of donation group or existence of siblings in the family.

**LIMITATIONS, REASONS FOR CAUTION:**

Participants were recruited from a multicenter longitudinal cohort study of families following donor conception and achieved a high response rate among adolescents, but we cannot exclude the possibility of attrition bias with less well-functioning families opting out of the longitudinal study over time. Also, despite a relatively large study sample, small subgroups limit statistical power, and results should therefore be regarded with caution. Finally, the study was conducted in Sweden with mandatory identity-release donation, limiting generalizability to other contexts.

**WIDER IMPLICATIONS OF FINDINGS:**

Among young people who are aware of their conception with identity-release donation from an early age, a majority intend to obtain information about the donor. Type of donation and quality of family relationships seem to have relevance for adolescents’ interest in seeking donor information, but more research is necessary. There appears to be a considerable variation regarding interest in different types of information, ranging from non-identifying characteristics to the donor’s identity and openness to contact. These findings suggest that release of donor information should be adapted to the individual wishes of the donor-conceived person.

**STUDY FUNDING/COMPETING INTEREST(S):**

This study was funded by the Swedish Research Council (grant number 2021-03174) and Umeå University. The authors have no conflict of interest to declare.

**TRIAL REGISTRATION NUMBER:**

N/A.

## Introduction

Over the past decades, there has been a steady increase in the number of heterosexual and lesbian couple as well as single women who use gamete donation to become parents. In the majority of cases, anonymous donors have been used, but more and more countries have adopted the praxis of identity-release donation where people conceived through gamete donation have the right to obtain the identity of the donor (Calhaz-Jorge *et al.*, 2024). In 2022, identity-release donation was available in 16 European countries (Calhaz-Jorge *et al.*, 2024), and a recent study showed an increase of identity-release sperm donors in the USA (Valido *et al.*, 2025). Following identity-release gamete donation, parents usually have no or limited non-identifying information about the donor, though there are differences between countries with regard to the information provided to the parents and the donor-conceived person (DCP) (Lukasiewicz, 2020; Calhaz-Jorge *et al.*, 2024). For example, in the Netherlands, the parents are provided with non-identifying information about the donor (e.g. medical history, vocation, hobbies) while in Sweden, the parents receive no donor information and only the DCP has the right to obtain information (Lukasiewicz, 2020). The identity of the donor is usually made available to the DCP when they reach adulthood (18 years of age) and in some countries, such as the UK, DCPs can access non-identifying information a few years earlier (Calhaz-Jorge *et al.*, 2024).

Sweden provides a unique example in an international context as it was the world’s first country to implement mandatory identity-release gamete donation in 1985 (Stoll, 2008). Treatment was initially only provided to heterosexual couples using donor insemination, but access to treatment has later been extended to heterosexual couples using IVF with donor oocytes or sperm (2003), as well as to lesbian couples (2005) and single women (2016) using sperm donation and in 2019, double donation and embryo donation became available to all groups. Matching between donors and recipients is conducted by clinicians based on physical characteristics, and no identifying or non-identifying donor information is provided to the prospective parents. Parents are encouraged to disclose both the donor conception and the child’s right to donor-information from an early age (The National Board of Health and Welfare, 2016). According to Swedish law (The Genetic Integrity Act 2006:351, 2006), DCPs are entitled to access the identity of the donor when sufficiently mature, which is typically interpreted as 18 years of age (Stenfelt *et al.*, 2023). Within the Swedish legislation, solely the DCP (not the parents) has the right to information and can thus access identifying and/or non-identifying donor information from the fertility clinic.

A systematic review including 47 studies published between 2003 and 2020 (Indekeu *et al.*, 2021) found that, across studies, donor-conceived persons (DCPs) expressed a strong interest in information about and/or contact with the donor. Commonly reported motives were curiosity regarding physical and behavioral resemblance, to learn about medical and genetic history, and to establish a relationship with the donor and same-donor peers. The review found that most studies recruited through online support forums or interest groups and concluded that there is a need to investigate interest in donor information among groups of DCPs that have not been self-selected due to their interest in the donor conception (Indekeu *et al.*, 2021). The authors also highlighted that previous research is largely focused on people conceived by anonymous sperm donation, and that less is known about the interests of DCPs with identifiable sperm donors and those conceived by donor oocytes.

In the USA, two research groups have surveyed adolescents and young adults conceived with identity-release sperm donation, using a longitudinal approach. Scheib *et al.* (2005) reported that a majority of adolescent DCPs were at least moderately interested in the identity of the donor and a third of young adult DCPs eligible for donor information had made such a request (Scheib *et al.*, 2017). Similarly, among DCPs with identity-release sperm donors in the “National Longitudinal Lesbian Family Study,” two-thirds stated at age 17 years that they planned to contact their donor (Bos and Gartrell, 2011) and a third had done so by age 25 years (Koh *et al.*, 2020). In comparison to DCPs in lesbian-couple and single-mother families, fewer of those in heterosexual-couple families planned to obtain donor information (Scheib *et al.*, 2005) and made such requests (Scheib *et al.*, 2017). Females were also more likely to request donor identity than males (Scheib *et al.*, 2017). In addition, qualitative results indicate that family dynamics, such as feeling different from one’s parents concerning academic achievements, and a poor relationship with the father (non-genetic parent), could prompt DCPs’ interest in obtaining information and establishing contact with the donor (Widbom *et al.*, 2024). In the study by Scheib *et al.* (2005) most adolescents with identifiable sperm donors also reported an interest in contact with other people conceived with sperm from the same donor, so-called “same-donor peers.”

Increasing numbers of DCPs following identity-release gamete donation are reaching an age when they can obtain non-identifying or identifying donor information, but little is known about their perspectives regarding seeking such information. In particular, there are no studies about the experiences and perceptions of those conceived with donor oocytes. Therefore, the overall aim of the present study was to investigate the perspectives of adolescents in lesbian-couple and heterosexual-couple families following identity-release sperm donation (SD-L, SD-H) or oocyte donation (OD-H), with a focus on their interest to request information about the donor. Specifically, we aimed to compare adolescents from these groups regarding disclosure issues, interest in donor-related information and contact, and to investigate variables associated with adolescents’ intention to request donor information, including family form (lesbian/heterosexual-couple) and donation type (sperm/oocyte donation).

## Materials and methods

### Study population

The present study was conducted as part of the longitudinal Swedish Study on Gamete Donation (SSGD) that consecutively recruited couples starting donation treatment at all university hospitals in Sweden during a 3-year period (2005-2008). Inclusion criteria were being part of a heterosexual or lesbian couple starting treatment with donor oocytes or sperm and being able to read and speak Swedish. Couples who did not undergo at least one round of treatment (i.e. insemination of donor sperm or transferral of ≥1 fertilized oocyte) were excluded. Out of 587 approached couples, a total of 450 couples were included; in all but five cases, both in the couple participated. Participants in the SSGD have been requested to complete individual postal surveys at several time points during the years; in total, we have conducted five waves of data collection. The fifth wave was conducted in 2022-2023 when the “target child” in the family (i.e. the first child born following treatment in 2005-2010) had reached adolescence (13-17 years). Couples who had not conceived a child as part of the study (n = 164) or had dropped out at previous waves of the SSGD were excluded from this data collection; for more details, see Paulin *et al.* (2024). Thus, a total of 216 families were approached at the fifth wave and parents in 165 of these families (76%) completed the parental survey. In conjunction with this, parents were asked for contact information to the target child, provided that the child was informed about his/her donor conception and right to request donor information. For children under 15 years of age, we also collected the parents’ informed consent for the child to participate in the study. Parents in 123 families (75%) provided contact information to the target child. In remaining 42 families, parents stated that they had not (yet) talked with the child about his/her donor conception (n = 9) or the possibility to obtain identifying donor information (n = 22), and some parents mentioned other circumstances related to the child (e.g. current mental state, neurodevelopmental disorder). In the case of twins, both were invited to participate. The study was approved by the Swedish Ethical Review Authority (2022-03739-01).

A total of 128 adolescents were approached, and 100 agreed to participate (78% response rate). After excluding four participants who had a known donor, the total sample for the present study consisted of 96 adolescents. A total of six twins participated: two full sets (one pair from a SD-L family and one pair from a SD-H family), and two single twins without their respective siblings (one SD-L and one SD-H).

Within the total sample of 96 adolescents (aged 13–16 years), about half were children in lesbian-couple families (N = 47, 49%), and the remainder in heterosexual-couple families following sperm (N = 27, 28%) or oocyte donation (N = 22, 23%) (Table 1). There was an even gender distribution within and between groups, but SD-H adolescents were significantly older than those in SD-L and OD-H families (*P *= 0.002). About two-thirds of the participants had married/cohabiting parents, with participants from SD-L families being more likely to have separated parents than those from heterosexual-couple families (*P *< 0.001). Most adolescents in the sperm donation families had siblings compared to OD-H adolescents that were significantly less likely to have any siblings compared to the other groups (*P < *0.001). Most adolescents had learned about their donor conception before the age of 8 years, with the highest percentages of early disclosure among those from lesbian-couple families (*P *= 0.007). The groups differed significantly regarding openness about the donor conception (*P *< 0.001); while almost all SD-L adolescents stated that both people within and outside the family know about their conception with donor sperm, a third of SD-H and OD-H adolescents reported that only their family members know about the donor conception.

**Table 1.** Background information on participants in lesbian- and heterosexual-couple families following sperm donation (SD-L, SD-H) and in oocyte donation families (OD-H).

	Total	SD-L	SD-H	OD-H	
	(*N *= 96)	(*N *= 47)	(*N *= 27)	(*N *= 22)	*P*-value
	N (%)	N (%)	N (%)	N (%)	
Gender					0.833^1^
Girl	47 (49.0)	22 (46.8)	13 (48.1)	12 (54.5)	
Boy	49 (51.0)	25 (53.2)	14 (51.9)	10 (45.5)	
Age (years)					0.002^1^^,^*
13-14	52 (54.2)	30 (63.8)	7 (25.9)	15 (68.2)	
15-16	44 (45.8)	17 (36.2)	20 (74.1)	7 (31.8)	
Parents’ relationship status					<0.001^1^^,^*
Married/co-habiting	58 (60.4)	20 (42.6)	23 (85.2)	15 (68.2)	
Divorced/separated	38 (39.6)	27 (57.4)	4 (14.8)	7 (31.8)	
Donor-conceived sibling(s)					<0.001^1^^,^*
Yes	56 (58.3)	32 (68.1)	19 (70.4)	5 (22.7)	
No	40 (41.7)	15 (31.9)	8 (29.6)	17 (77.3)	
Age at disclosure					0.007^2^^,^*
Very young/always known	65 (67.7)	37 (78.7)	17 (63.0)	11 (50.0)	
7 years or younger	16 (16.7)	8 (17.0)	2 (7.4)	6 (27.3)	
8 years or older	15 (15.6)	2 (4.3)	8 (29.6)	5 (22.7)	
People who know about the donor conception (besides parents)					<0.001^2^^,^*
Both family and others^3^	68 (70.8)	43 (91.5)	14 (51.9)	11 (50.0)	
Only family	18 (18.8)	1 (2.1)	10 (37.0)	7 (31.8)	
Only others^3^	6 (6.3)	2 (4.3)	2 (7.4)	2 (9.1)	
No one	3 (3.1)	1 (2.1)	1 (3.7)	1 (4.5)	
Missing	1 (1.0)	–	–	1 (4.5)	

1 Pearson χ^2^ test.

2 Fisher’s exact test.

3 E.g. friends, partner.

* *P *< 0.05.

### Data collection and measurements

Adolescents were approached with a postal survey including study information, an informed consent sheet, a paper survey, and a link to a web version of the survey. Two reminders were sent, and participants received two movie tickets; for further details about the recruitment of adolescents, please see Groundstroem *et al.* (2025). The survey was distributed in two versions using parental terms relevant for adolescents in lesbian-couple families (Birth mother and Mother who did not give birth to me) and heterosexual-couple families (Mother and Father). To facilitate presentation of methods and results, the terms “Mother” (indicating birthmother) and “Co-parent” are used for data from all groups.

#### Sociodemographic variables

Participant characteristics included donation group (SD-L, SD-H, OD-H), age, gender (Girl, Boy, Other, Decline to state), parents’ relationship status (Married/co-habiting, Divorced/separated), and having donor-conceived siblings in the family (Yes, No).

#### Disclosure issues

Participants were requested to report the age when they learned of their donor conception (≤7 years, ≥8 years, Very young/always known). In addition, they were asked to state which persons know about their origin with donor conception with six response alternatives (No one besides my parents, Siblings, Other members of my family, Girlfriend/Boyfriend/Partner, Friends, Others). Responses were collapsed into four categories describing groups that know about the donor conception, besides the parents: Both family and others, Only family, Only others (e.g. friends, partner), No one.

#### Interest in donor information and contact with same-donor peers

The section of the survey measuring interest in donor information included a short introductory paragraph stating that all DCPs in Sweden have the right to know who their donor is, and that DCPs at 18 years of age can contact the fertility clinic to obtain information about their donor. Intention to request information about the donor was then assessed with the question “Do you plan to search for information about your donor?” with four response alternatives (Yes, as soon as possible, Yes, sometime in the future, Uncertain, No). In the regression analyses, the two positive alternatives were collapsed, resulting in three response categories (Yes, Uncertain, No).

All, except those who responded “No,” were requested to respond to three follow-up questions. Motivations to request donor information were assessed with six response alternatives (Physical resemblance, Resemblance of non-physical traits, Information about heritage, Information about medical background, Relevance to own identity, Contact with donor) used in a previous study with young adult DCPs (Lampic *et al.*, 2022). Donor information desired from the clinic was assessed with five response alternatives (Background information, e.g. age and education, Name and personal identity number, Contact information, If donor accepts to be contacted, Other). Participants were also asked to state if they had talked with their parents about their intention to request donor information (Yes, mother, Yes, co-parent, Yes, both parents, No). Finally, all participants were requested to state if they are interested in contact with other people conceived with gametes from the same donor (Yes, No, Uncertain).

#### Family relationships

Family functioning was assessed by a short version of the General Functioning Subscale based on the McMaster Family Assessment Device (Epstein *et al.*, 1983). The short version (FAD GF6+) includes the six positive items from the GF12 subscale, e.g. “In times of crisis, we can turn to each other for support.” Responses are indicated on a four-point Likert scale, with higher mean scores indicating worse functioning and a mean score of 2 suggested as cut-off to separate healthy from unhealthy family functioning (Boterhoven de Haan *et al.*, 2015). The GF6+ is a reliable and valid instrument to assess overall family functioning and has demonstrated equivalent psychometric properties to the GF12 (Boterhoven de Haan *et al.*, 2015). Internal consistency in the present study was high (α  = 0.80). The distribution was moderately to highly positively skewed (skewness = 1.03), showing that most adolescents rated healthy family functioning.

Aspects of the parent-child relationship were assessed by asking adolescents to rate the perceived emotional closeness and resemblance (physical and non-physical characteristics) with both parents. Emotional closeness was assessed with a single item “Overall, how close would you say you are to your [mother/co-parent]?” with responses indicated on a four-point Likert-scale (Not at all close, Not very close, Fairly close, Very close). Perceived resemblance of physical and non-physical characteristics was assessed by two study-specific items: “How much do you think you resemble your [mother/co-parent] (a) in appearance, (b) in the way you are?”. Responses were indicated on a four-point Likert-scale (Not at all, Somewhat, Quite a lot, Very much). In the analyses, the responses were dichotomized based on distributions: for perceived closeness to parents (Very close vs All other responses) and for perceived resemblance with parents (Very much and Quite a lot vs Somewhat and Not at all).

### Data analysis

Differences in background characteristics and interest in donor-related information and contact between the three groups of adolescents (SD-L, SD-H, OD-H) were analyzed with Pearsons χ^2^ or Fishers’ exact test. Multinomial logistic regression analysis was used to analyze variables associated with adolescents’ intention to request donor information (Yes, Uncertain, No). First, the variables hypothesized to be associated with the outcome were analyzed individually using univariate multinomial logistic regression analysis. The following variables were included: gender (Girl/Boy), family form (Lesbian/Heterosexual-couple), heterosexual-couple donation group (SD-H/OD-H), age at disclosure (≤7 years/≥8 years), family function (Continuous), as well as closeness (Very close/All other responses), physical resemblance, and resemblance of non-physical characteristics (Very much and Quite a lot/Not so much and Not at all), separately for the genetic parent and the non-genetic parent. All variables showed low collinearity (VIF ≤1.3), indicating that they were not correlated with each other and two of the variables (family function and closeness with non-genetic parent) were found to be significantly associated with the outcome. Next, multinomial logistic regression analysis was conducted in two separate models. The first model included the two variables individually associated with the outcome, as well as the variables family form (Lesbian/Heterosexual) and gender (Girl/Boy) based on previous research results. The second model was restricted to the subsample of participants from heterosexual-couple families (N = 49) and included the two variables individually associated with the outcome, as well as the variables donation group (SD-H/OD-H) and gender (Girl/Boy). For the outcome, responding “Yes” regarding the intention to request donor information was used as the reference category. All statistical analysis were conducted using IBM SPSS Statistics for Windows, version 28 and 30 (IBM Corp., Armonk, NY, USA). A *P*-value of < 0.05 was considered statistically significant.

## Results

### Interest in donor information and contact with donor and same-donor peers

More than half of the participants stated that they planned to request (any) donor information as soon as possible or sometime in the future (Table 2). A third of the adolescents were uncertain and about 1 in 10 did not intend to request donor information. Desired information about the donor included both identifying and non-identifying information. A majority of adolescents reported that they want to receive information about the donor’s background (78%) and identity (61%), and about half would like to know the donor’s stance regarding potential contact (Table 2). Approximately a third (35%) wanted contact information to the donor. The most common motivations for intending to seek donor information were to look for resemblance in appearance and non-physical traits, and to learn more about heritage and medical history; one in five stated contacting the donor as a motivation. There were no significant differences between the donation groups regarding motivating factors or desired information (Table 2).

**Table 2.** Interest in donor information and same-donor peers among adolescent DCPs in lesbian- and heterosexual-couple families following sperm donation (SD-L, SD-H) and oocyte donation (OD-H), by donation group.

	Total	SD-L	SD-H	OD-H	
	(*N *= 96)	N = 47	N = 27	N = 22	*P*-value
	N (%)	N (%)	N (%)	N (%)	
Intention to request donor information					0.095^1^
Yes, as soon as possible	27 (28.1)	17 (36.2)	5 (18.5)	5 (22.7)	
Yes, sometime in the future	29 (30.2)	11 (23.4)	7 (25.9)	11 (50.0)	
Uncertain	31 (32.3)	16 (34.0)	12 (44.4)	3 (13.6)	
No	9 (9.4)	3 (6.4)	3 (11.1)	3 (13.6)	
Motivation for requesting donor information^3^^,^^4^					
Physical resemblance	62 (71.3)	31 (70.5)	18 (75.0)	13 (68.4)	0.906^2^
Resemblance of non-physical characteristics	46 (52.9)	26 (59.1)	13 (54.2)	7 (36.8)	0.280^2^
Information about heritage	56 (64.4)	31 (70.5)	14 (58.3)	11 (57.9)	0.534^2^
Information about medical background	39 (44.8)	21 (47.7)	9 (37.5)	9 (47.4)	0.720^2^
Relevance to own identity	21 (24.1)	15 (34.1)	4 (16.7)	2 (10.5)	0.088^2^
To contact donor	19 (21.9)	12 (27.3)	3 (12.5)	4 (21.1)	0.387^1^
Other	14 (16.1)	8 (18.2)	4 (16.7)	2 (10.5)	0.863^1^
Desired donor information from the clinic^3^^,^^4^					
Background information	68 (78.2)	35 (79.5)	18 (75.0)	15 (78.9)	0.941^1^
Name, personal identity number	53 (60.9)	28 (63.6)	14 (58.3)	11 (57.9)	0.878^2^
Contact information	31 (35.6)	16 (36.4)	7 (29.2)	8 (42.1)	0.671^2^
If donor accepts to be contacted	45 (51.7)	22 (50.0)	13 (54.2)	10 (52.6)	0.960^2^
Other	15 (17.2)	10 (22.7)	3 (12.5)	2 (10.5)	0.427^1^
Talked with parents about requesting donor information^3^					0.012^1^^,^*
Yes, both parents	51 (58.6)	32 (72.7)	12 (50.0)	7 (36.8)	
Yes, mother	9 (10.3)	1 (2.3)	2 (8.3)	6 (31.6)	
Yes, co-parent	2 (2.3)	–	1 (4.2)	1 (5.3)	
No	19 (21.8)	8 (18.2)	6 (25.0)	5 (26.3)	
Other	3 (3.4)	1 (2.3)	2 (8.3)	–	
Missing	3 (3.4)	2 (4.5)	1 (4.2)	–	
Interest in contact with same-donor peers					0.945^2^
Yes	29 (30.5)	15 (32.6)	7 (25.9)	7 (31.8)	
Uncertain	42 (44.2)	21 (45.7)	12 (44.4)	9 (40.9)	
No	24 (25.3)	10 (21.7)	8 (29.6)	6 (27.3)	
Missing	1 (1.0)	1 (2.1)	–	–	

1 Fisher’s exact test.

2 Pearson χ^2^ test.

3 Item completed by a total of 87 participants, i.e. not by those who responded “No” regarding intention to request donor information.

4 Multiple responses possible.

* *P *< 0.05.

Participants differed significantly regarding involving their parents in conversations about their intention to request donor information (*P *= 0.012) (Table 2). While most adolescents in lesbian-couple families had talked with both parents, this was less common among those in heterosexual-couple families.

A third of the adolescents expressed an interest in connecting with same-donor peers, almost half were uncertain and the remainder stated no interest (Table 2). There was no significant difference between donation groups, nor between adolescents with and without donor-conceived siblings (*P *= 0.601).

### Variables associated with adolescents’ intention to request donor information

Potential explanatory variables and their relation to adolescents’ intention to request donor information are presented in Table 3. All explanatory variables were individually analyzed using univariate multinomial logistic regression, with three of them being significantly associated with being uncertain versus intending to request donor information: family function (OR 0.14, 95% CI 0.03-0.61), closeness with the non-genetic parent (OR 0.36, 95% CI 0.14-0.95), and donation group within heterosexual-couple families (OR 0.19, 95% CI 0.04-0.82).

**Table 3.** Adolescents’ intention to request donor information in relation to selected sociodemographic and psychosocial factors—odds ratios (OR) and CIs for individual associations.

	Intention to request donor information			Univariate multinomial logistic regression analysis	
	Yes (N = 56)	Uncertain (N = 31)	No (N = 9)	Uncertain^1^	No^1^
	*M (SD)*	*M (SD)*	*M (SD)*	*OR (CI 95%)* ^2^	*OR (CI 95%)* ^2^
Family functioning (FAD GF6+)^3^	1.46 (0.40)	1.22 (0.31)	1.26 (0.36)	0.14 (0.03, 0.61)*	0.21 (0.02, 1.96)
	*N (%)*	*N (%)*	*N (%)*		
Family form					
Lesbian-couple	28 (50)	16 (51.6)	3 (33.3)	1.07 (0.44, 2.57)	0.50 (0.11, 2.20)
Heterosexual-couple	28 (50)	15 (48.4)	6 (66.7)	Ref	Ref
Donation group^4^					
OD-H	16 (57.1)	3 (20)	3 (50)	0.19 (0.04, 0.82)*	0.75 (0.13, 4.39)
SD-H	12 (42.9)	12 (80)	3 (50)	Ref	Ref
Gender					
Girl	24 (42.9)	17 (54.8)	6 (66.7)	1.62 (0.70, 3.92)	2.67 (0.61, 11.76)
Boy	32 (57.1)	14 (45.2)	3 (33.3)	Ref	Ref
Age at disclosure^5^					
7 years or younger	46 (82.1)	28 (90.3)	7 (77.8)	2.03 (0.51, 8.01)	0.76 (0.14, 4.22)
8 years or older	10 (17.9)	3 (9.7)	2 (22.2)	Ref	Ref
Closeness to genetic parent^6^					
Very close	37 (67.3)	24 (77.4)	7 (87.5)	0.60 (0.22, 1.65)	0.29 (0.03, 2.57)
Other responses	18 (32.7)	7 (22.6)	1 (12.5)	Ref	Ref
Closeness to non-genetic parent^6^					
Very close	28 (50.9)	23 (74.2)	6 (75)	0.36 (0.14, 0.95)*	0.35 (0.06, 1.87)
Other responses	27 (49.1)	8 (25.8)	2 (25)	Ref	Ref
Physical resemblance with genetic parent^6^					
Very/quite much	32 (58.2)	18 (60.0)	7 (87.5)	0.93 (0.38, 2.30)	0.20 (0.02, 1.73)
Other responses	23 (41.8)	12 (40)	1 (12.5)	Ref	Ref
Physical resemblance with non-genetic parent^6^					
Very/quite much	7 (12.7)	4 (12.9)	1 (11.1)	0.98 (0.26, 3.67)	0.89 (0.13, 10.80)
Other responses	48 (87.3)	27 (87.1)	8 (88.9)	Ref	Ref
Resemblance of non-physical characteristics with genetic parent^6^					
Very/quite much	40 (80)	23 (76.7)	6 (75)	1.21 (0.41, 3.63)	0.75 (0.23, 7.63)
Other responses	10 (20)	7 (23.3)	2 (25)	Ref	Ref
Resemblance of non-physical characteristics with non-genetic parent^6^					
Very/quite much	25 (47.2)	15 (50)	7 (77.8)	0.89 (0.36, 2.18)	0.26 (0.05, 1.34)
Other responses	28 (52.8)	15 (50)	2 (22.2)	Ref	Ref

1 Reference category: Yes.

2 Odds ratio (OR) and CI.

3 Total mean score, from 1 (best functioning) to 4 (worse functioning).

4 Analysis includes only heterosexual-couple family following sperm donation (N = 27 SD-H) and heterosexual-couple family following oocyte donation (N = 22 OD-H).

5 Age at disclosure, dichotomized by including “Very young/always known” with the category “7 years or younger.”

6 Due to missing values, the total number of responses to these variables varied between 88 and 95.

* *P *< 0.05.

The association between selected explanatory variables and the outcome variable “Intention to request donor information” was analyzed in two different multinomial regression models, with Yes as reference category (Table 4). The first model included four explanatory variables (family form, gender, family function, and closeness with non-genetic parent). The only significant association found was for family functioning (FAD GF6+) with the outcome category Uncertain (*P *= 0.036) (OR 0.19, 95% CI 0.04–0.90). The results showed that adolescents with better family functioning were less likely to intend requesting donor information and more likely to be unsure. The second model was restricted to adolescents from heterosexual-couple families and included four explanatory variables (donation group, gender, family function, and closeness with non-genetic parent). Donation group was the only variable in this model significantly associated with the outcome category (*P *= 0.028) (OR 0.17, 95% CI 0.04–0.82) and showed that adolescents in SD-H families were less likely to intend requesting donor information and more likely to be unsure compared to those in OD-H families. No variables were found to significantly predict the intention to not request donor information in either of the models.

**Table 4.** Multivariable multinomial logistic regression models of the relationship between adolescents’ intention to request donor information and four predictors—conducted separately for family forms (total sample, n = 96) and type of donation (heterosexual-couple families, n = 49).

Intention to request donor information	Uncertain^1^ Parameter estimates		No^1^ Parameter estimates	
	Sig	OR (CI 95%)	Sig	OR (CI 95%)
Total sample (N = 96)				
Family form				
Lesbian-couple	0.759	0.86 (0.32, 2.29)	0.402	0.50 (0.10, 2.50)
Heterosexual-couple	Ref		Ref	Ref
Gender				
Girl	0.171	1.97 (0.75, 5.23)	0.217	2.73 (0.55, 13.45)
Boy	Ref		Ref	Ref
Family function (FAD GF6+)	0.036*	0.19 (0.04, 0.90)	0.392	0.37 (0.04, 3.57)
Closeness, non-genetic parent				
Very close	0.136	2.33 (0.77, 7.11)	0.252	2.90 (0.47, 17.99)
All other responses	Ref		Ref	Ref
Heterosexual-couple families (N = 49)				
Donation group^2^				
OD-H	0.028*	0.17 (0.04, 0.82)	0.950	1.07 (0.12, 9.23)
SD-H	Ref		Ref	
Gender				
Girl	0.186	2.72 (0.62, 11.92)	0.308	3.06 (0.36, 26.30)
Boy	Ref		Ref	
Family function (FAD GF6+)	0.437	0.41 (0.04, 3.95)	0.191	0.03 (0.00, 5.73)
Closeness, non-genetic parent				
Very close	0.756	1.28 (0.27, 6.04)	0.598	2.09 (0.14, 32.01)
All other responses	Ref		Ref	

1 Reference category: Yes.

2 Heterosexual-couple family following sperm donation (SD-H), Heterosexual-couple family following oocyte donation (OD-H).

* *P *< 0.05.

## Discussion

In this long-term follow-up of lesbian- and heterosexual-couple families following identity-release gamete donation, just over half of donor-conceived children (aged 13–16 years) intended to request some information about the donor, either at legal age or sometime in the future. This intention was not related to gender or being raised in a lesbian-couple or heterosexual-couple family. However, those reporting better family functioning were more likely to be uncertain about seeking donor information. Among adolescents in heterosexual-couple families, those conceived with donor oocytes were significantly more interested in requesting donor information, while those conceived with sperm donation were more likely to be uncertain. At the time of the study, a majority, but not all, stated that they wanted to obtain the donor’s identity and half would like to know if the donor is open to being contacted. Interest in contact with the donor and same-donor peers was reported by a minority, with no difference between adolescents from different types of families.

Our results that ∼60% of the adolescents intended to request some donor information, half of which intended to do so “as soon as possible” (ASAP), are largely in line with previous findings from two longitudinal studies of families using identity-release sperm donation (Scheib *et al.*, 2005, 2017; Bos and Gartrell, 2011; Koh *et al.*, 2020). As these studies have shown a considerable discrepancy between intending to seek donor information or contact and actually doing so, it is unclear how many of the present study participants will request information about their donor when able to do so (at 18 years of age), calling for a follow-up of how this develops over time.

In contrast to previous findings (Scheib *et al.*, 2005, 2017), participants’ interest in donor information was not related to family form or gender. Instead, we found that adolescents that rated better family functioning were less likely to intend seeking donor information and more likely to be unsure about this. Previous research on the current sample has shown that adolescents rating higher scores on security of attachment to the co-parent were less curious about the donor conception (Groundstroem *et al.*, 2025), whereas a study by Slutsky *et al.* (2016) has shown an opposite pattern, with securely attached adolescents expressing higher degrees of donor curiosity compared to those with an insecure attachment. The inconsistent results suggest that the relationship between family functioning and donor curiosity is complex and likely associated with other factors as well, such as concerns that searching for and contacting the donor could affect parents negatively (Jadva *et al.*, 2010; Beeson *et al.*, 2011; Lampic *et al.*, 2022). Thus, being uncertain about seeking donor information among those with better family functioning may not necessarily represent a lower interest but could be influenced by fear of harming good relations, or reflect not having thought much about the possibility of obtaining information about the donor. Finally, it should be noted that the adolescents in the present study overall had ratings on the FAD GF6+ that indicated healthy family functioning, including among the group that intended to request donor information.

The present study was the first to assess intention to obtain donor information among adolescents conceived by identity-release oocyte donation and found that a large majority (>70%) in this group planned to do so. This group was significantly more likely to express an intention to request donor information compared to those conceived with donor sperm to heterosexual couples, who were more likely to be uncertain in this regard. One possible explanation for this finding is that the latter group worried that searching for the donor could have a negative impact on the relationship with their (non-genetic) father, as previously reported (Jadva *et al.*, 2010; Beeson *et al.*, 2011). In oocyte donation families, the non-genetic parent is the birth mother, who has a biological bond to the child through pregnancy and breast-feeding. Having a gestational bond with the child, despite not having a genetic link, has been shown to be important in establishing feelings of motherhood in same-sex mothers using reciprocal IVF (Bower-Brown *et al.*, 2024). Mothers through oocyte donation have also been shown to pursue active strategies to cope with their feelings regarding the donation and to define their parental roles (Imrie *et al.*, 2020; Lysons *et al.*, 2022), helping them to feel secure as mothers (Imrie *et al.*, 2020). Such processes may contribute to a mother-child bond perceived as sufficiently strong not to be threatened by the DCP’s interest in the oocyte donor. In fact, one-third of adolescents conceived with donor oocytes had discussed their intentions regarding obtaining donor information only with their mother, while few of the other participating adolescents had talked about this with only one of their parents. In heterosexual-couple families following sperm donation, mothers tend to be more open to talking about donation issues compared to fathers who are often reported to avoid the subject (Beeson *et al.*, 2011; Widbom *et al.*, 2021; Cosson *et al.*, 2022), assumably due to the stigma associated with male infertility (Wischmann and Thorn, 2013). As most conducted studies concern sperm donation, there is a need for more research on communication patterns in oocyte donation families.

A common motivator for requesting donor information is to look for resemblance in appearance or non-physical traits with the donor (Scheib *et al.*, 2017; Indekeu *et al.*, 2021; Lampic *et al.*, 2022; Widbom *et al.*, 2024) and this was also found in the present study. As could be expected, participants overall thought they resembled the genetic parent more than the non-genetic parent, but perceived resemblance (or lack thereof) was not found to be associated with the intention to request donor information. In contrast to previous studies of DCPs in heterosexual-couple families that have indicated higher interest in donor information among females (Scheib *et al.*, 2017; Lampic *et al.*, 2022), we did not find an effect of gender on adolescents’ intention to obtain information about the donor.

When asked what type of donor information adolescents would like to receive from the clinic, most stated that they wanted general non-identifying information, such as the donor’s age and occupation. Many, but not all, wanted to know the donor’s identity and half wanted to know whether the donor is open to contact. A desire for information about the donor’s attitude toward contact has previously been reported by adolescent and young adult DCPs (Scheib *et al.*, 2005; Lampic *et al.*, 2022). It also aligns with the recent finding that Swedish identity-release gamete donors would like clinics to convey their stance on potential contact to offspring from their donations (Lampic *et al.*, 2025). The present results of varying interest in obtaining specific information about the donor are reflected in the Swedish practice guidelines for identity release (Stenfelt *et al.*, 2023), which emphasize that the amount and type of provided information must be adapted to the DCP’s individual wishes.

About a third of participating adolescents expressed an interest in contact with same-donor peers, with no differences between family types or between those with and without siblings. This number is remarkably lower compared to the adolescents in the study by Scheib *et al.* (2005), where almost 90% were at least moderately interested in contact with same-donor peers. It is possible that this reflects a more general difference between the samples regarding their interest in donation-related issues, with the participants of the current study reporting relatively low levels of curiosity about their donor conception (Groundstroem *et al.*, 2025). In addition, the parents of the adolescents studied by Scheib *et al.* (2005) had actively chosen identity-release sperm donation, suggesting that they regarded the prospect of contact with the donor and same-donor peers more positively compared to the Swedish parents for whom identity-release donation was obligatory. Parental perspectives and family dynamics may affect DCPs’ information and contact-seeking behaviors, warranting further research.

The present study has methodological strengths and limitations that are important to consider. One strength of the present study is that it is one of the first studies to incorporate the views and intentions of adolescents conceived through identity-release oocyte donation, allowing group comparisons based on donation and family type. Another strength is that the study sample was part of the longitudinal multicenter Swedish Study on Gamete Donation and, as such, can provide a better representation of DC adolescents’ interest in gaining knowledge about their donor origins than studies based on self-selected participants with a particular interest in the subject of donor conception. While this is likely to reduce sampling bias, longitudinal studies carry with them a risk of systematic dropout of families who are less well-adjusted. In addition, when recruiting participants for the present study, we were reliant on the parents having disclosed the donor conception to the child and consenting to us contacting their child. The 123 families that provided contact information to their donor-conceived adolescent child for the present study represent about a fourth of the 450 couples that initially entered the SSGD and approximately a fifth of all couples that started donation treatment in Sweden during the 3-year recruitment period (2005-2008). As many couples in the SSGD did not conceive a child, the participating adolescents in the current study represent about a third of all children born within SSGD, and likely a significant portion of all DCPs born of treatment in Sweden during this time-period. Out of the predictor variables that individually were significantly associated with adolescents’ intention to request donor information, only two remained significant in the final regression models: family functioning and donation group, respectively. This may partly be explained by insufficient statistical power. While our study sample was relatively large compared to previous unselected samples of young people following identity-release donation (Scheib *et al.*, 2005; Bos and Gartrell, 2011), the subgroups become small when breaking them down for analysis. This is especially true for the adolescent group who did not intend to seek donor information, where the limited number of participants (n = 9) means that the results of the analysis comparing this group with the others should be interpreted with caution. Another limitation is our use of study-specific items to assess disclosure issues, interest in donor information and contact, and aspects of family relationships. These items have not been validated but they are based on previous research regarding perceptions and experiences of requesting information about identity-release donors (Scheib *et al.*, 2017; Lampic *et al.*, 2022; Widbom *et al.*, 2024).

The present findings showed considerable variation in adolescents’ intention to request identifying information about, and contacting, the donor and contribute to the recent discussions on abolishing legal age limits for access to donor information. While Swedish legislation gives the donor-conceived child the exclusive right to obtain identifying information about the donor when 18 years of age (Stoll, 2008), abolishing the legal age limit for access to donor information has been argued to be in the best interest of the child as it enables parents to tailor information and potential contact with donor or same-donor peers to the child’s needs (Bolt *et al.*, 2024; Indekeu *et al.*, 2025). Others have pointed to potential complications related to the role of the non-genetic parent and the donor, maintaining family boundaries, and the autonomy of the donor-conceived child (Pennings, 2024; Gilman *et al.*, 2025). In addition, previous research has found considerable variation regarding adolescent and young adult DCPs’ interest in searching for and contacting the donor (Zadeh *et al.*, 2018; Lampic *et al.*, 2022; Jadva *et al.*, 2023) even among donor-conceived siblings within the same family (Lampic *et al.*, 2022; Duff and Goedeke, 2025). Furthermore, findings from a recent study suggest that young adult DCPs in the UK are in support of the current national legislation of identity-release at 18 years of age as it keeps the deciding power with the DCP (Young Adults Study Report, n.d.).

In conclusion, identity-release gamete donation was introduced to provide DCPs with the opportunity to access information about their genetic origins, but there is limited knowledge about the demand for such information. The present study is based on a long-term follow-up of lesbian- and heterosexual-couple families within a Swedish multicenter study and was one of the first to include adolescents conceived with donor oocytes. We found that, among young people who were aware of their conception with identity-release donation from an early age, just over half intended to obtain information about the donor and a third were uncertain. Type of donation and quality of family relationships seem to have relevance for adolescents’ interest in seeking donor information but more research is necessary, especially to explore reasons for the quite high number of adolescents being uncertain about requesting donor-information. There appears to be a considerable variation regarding interest in different types of information, ranging from non-identifying characteristics to the donor’s identity and openness to contact. These findings suggest that release of donor information should be adapted to the individual wishes of the donor-conceived person. Finally, our results regarding adolescents’ interest in information about and contact with the oocyte donor warrant further exploration, preferably with qualitative methods.

## Data Availability

The data underlying the article will be shared on reasonable request to the corresponding author.
